# The Iontronic Quantum Dot

**DOI:** 10.1021/acs.nanolett.5c03173

**Published:** 2025-11-27

**Authors:** Domenic Prete, Valeria Demontis, Valentina Zannier, Lucia Sorba, Fabio Beltram, Francesco Rossella

**Affiliations:** † NEST, 19004 Scuola Normale Superiore and Istituto Nanoscienze-CNR, Piazza San Silvestro 12, I-56127 Pisa, Italy; ‡ Dipartimento di Scienze Fisiche, Informatiche e Matematiche, Università di Modena e Reggio Emilia, via Campi 213/a, 41125 Modena, Italy

**Keywords:** Iontronic Quantum Dot, Ion Gating, Solid-State Quantum Technologies

## Abstract

Semiconductor quantum dots (QDs) are key building blocks for quantum technologies with applications in quantum computation, communication, and sensing. QD device architectures rooted in conventional solid-state device fabrication paradigms are grappled with complex protocols to balance ease of realization, scalability, and transport properties. Using ion gating, we demonstrate a novel paradigm of quantum device engineering, that enables the realization and control of the *iontronic* QD. Clear Coulomb blockade peaks and their dependence on an externally applied magnetic field are reported, together with the impact of device architecture and confinement potential on QD quality. Devices incorporating two identical iontronic QDs in series are realized, addressing the reproducibility of the approach. A novel class of zero-dimensional quantum devices, iontronic QDs, overcome the need for thin dielectric layers, facilitating single-step device fabrication. This approach holds the potential to impact the development of quantum materials and devices in the context of solid-state quantum technologies.

## Graphical abstract

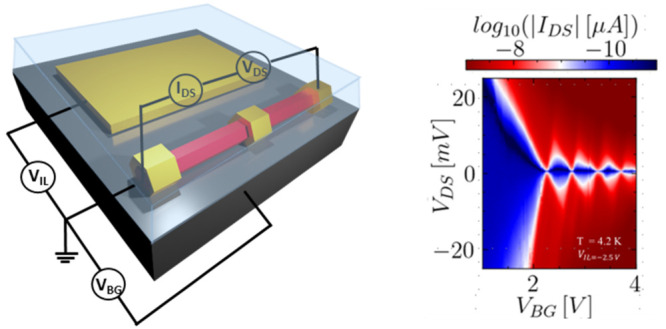

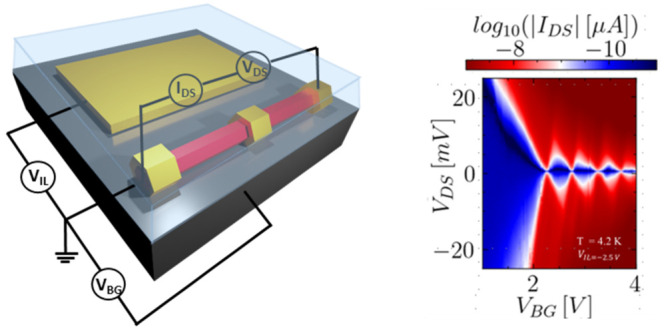

Quantum technologies, enabling technology and top priority for research and industry, potentially offer unprecedented advancements that might significantly impact society.^1,2^ The development of better-performing quantum platforms, allowing to study physical phenomena at the fundamental quantum level, is essential to unlocking new opportunities,^3^ and progress in quantum technologies is leveraged on the extensive expertise in nanoelectronics with semiconducting materials. In this framework, Quantum Dots (QDs) are crucial to implement Single Electron Transistors (SETs), serving as the fundamental building blocks for semiconductor-based quantum computing.^4–7^ To this aim, hard-wall and electrostatically defined QDs were realized in both CMOS compatible silicon architectures^8^ and III–V semiconductors.^9–12^

However, semiconductor-based quantum technologies are struggling to gain momentum compared to counterparts based on other materials,^13^ and despite the substantial progress achieved in the development of high-quality semiconductors, device fabrication has largely leaned toward conventional techniques based on the standard MOSFET architecture. Novel ideas and approaches might emerge from recent advancements in device nanoelectronics, which have capitalized on the methodologies of iontronics.^14–16^ In this, ultrastrong electric fields are applied conformally at the interface between a semiconductor and an electrolyte—ionic liquid, polymeric or ceramic electrolyte—by controlling the spatial distribution of ions in the electrolyte and by inducing ionic accumulation on the semiconductor’s surface.^17,18^ Iontronics devices offer unique advantages: maximized gate capacitance due to the electrolyte–semiconductor direct contact, high robustness of electrolytes against dielectric breakdown, electrical-noise reduction.^19–21^ These make ion gating a promising technique for providing nanoscale gates in quantum devices, enabling innovative experimental platforms for semiconductor-based quantum technologies.^22,23^

Here we report for the first time the implementation of a QD by combining a one-dimensional nanostructure (homogeneous InAs nanowire) and an electrolyte ([Emim]­[Tf2N] ionic liquid). The unprecedented electric field intensities accessible by ion gating are leveraged to define a zero-dimensional region in the nanowire by charge depletion mode, achieved thanks to a screening patch fabricated on the nanowire, which locally prevents depletion in the semiconductor section underneath, defining a zero-dimensional system. This is implemented in a dual-gate device realized with a single fabrication step, avoiding the multiple-step fabrication protocols needed to realize electrostatically defined solid-state QD devices using thin oxide layers as gate dielectrics.^24,25^ Ultimately, this allows us to develop a novel structure—the *iontronic quantum dot* (iQD). Magneto-transport at 4.2 K reveals a Coulomb blockade and allows the confinement features of the iQD to be characterized, which exhibit excellent performances including Coulomb oscillations with a contrast comparable to that of hard-wall QDs defined in heterostructure nanowires. The parameter space of the iQD is mapped computationally, using finite-element analysis, and experimentally, fabricating devices with different screening-patch widths and measuring Coulomb blockade in different gating configurations. Furthermore, we test our approach on the realization of two nominally identical iQDs connected in series, probing their energy spectrum. Notably, the specific technology used for patterning—electron beam lithography in the present case—does not represent a determining factor for the operation of the device. Other technologies such as (3D) nanoimprinting, extreme UV lithography and others can in principle be employed, if a patterning resolution of the order of tens of nanometer is achieved.

Our results show a new pathway to tailor quantum nanostructures by combining the vast technological know-how on semiconductors and the high-performance of ion gating. The spectrum of potential applications of the newly developed quantum systems is very wide and includes novel ways to implement spin qubits and to explore quantum thermodynamics on complex nanoscale systems.

The device architecture is schematically shown in Figure 1. A homogeneous InAs nanowire (2 μm long, 80 nm diameter) is drop-casted onto a Si++/SiO_2_ substrate allowing to apply the back-gate voltage *V*_BG_ and is contacted with two Ohmic contacts for voltage biasing *V*_DS_ and for the measurement of the drain-to-source current *I*_DS_ (see Supporting Information S. Methods for further details on nanowire growth and device fabrication). Completing the device design is a metallic patch positioned between the two contacts, as depicted in the inset of Figure 1(a). An actual device is shown in Figure 1(b), which reports a tilted scanning electron micrograph showing the electrical contacts, the nanowire, and the 100 nm wide metal patch. The entire device is embedded in a droplet of ionic liquid ([Emim]­[Tf2n]) whose ionic arrangement can be driven by applying a voltage *V*_IL_ to a planar counter-electrode. Noticeably, this device architecture is fabricated with a single lithographic step and allows for dual-gate operation^20^ thanks to the simultaneous presence of the Si++/SiO_2_ back-gate and the ionic-liquid gate.

**Figure 1:**
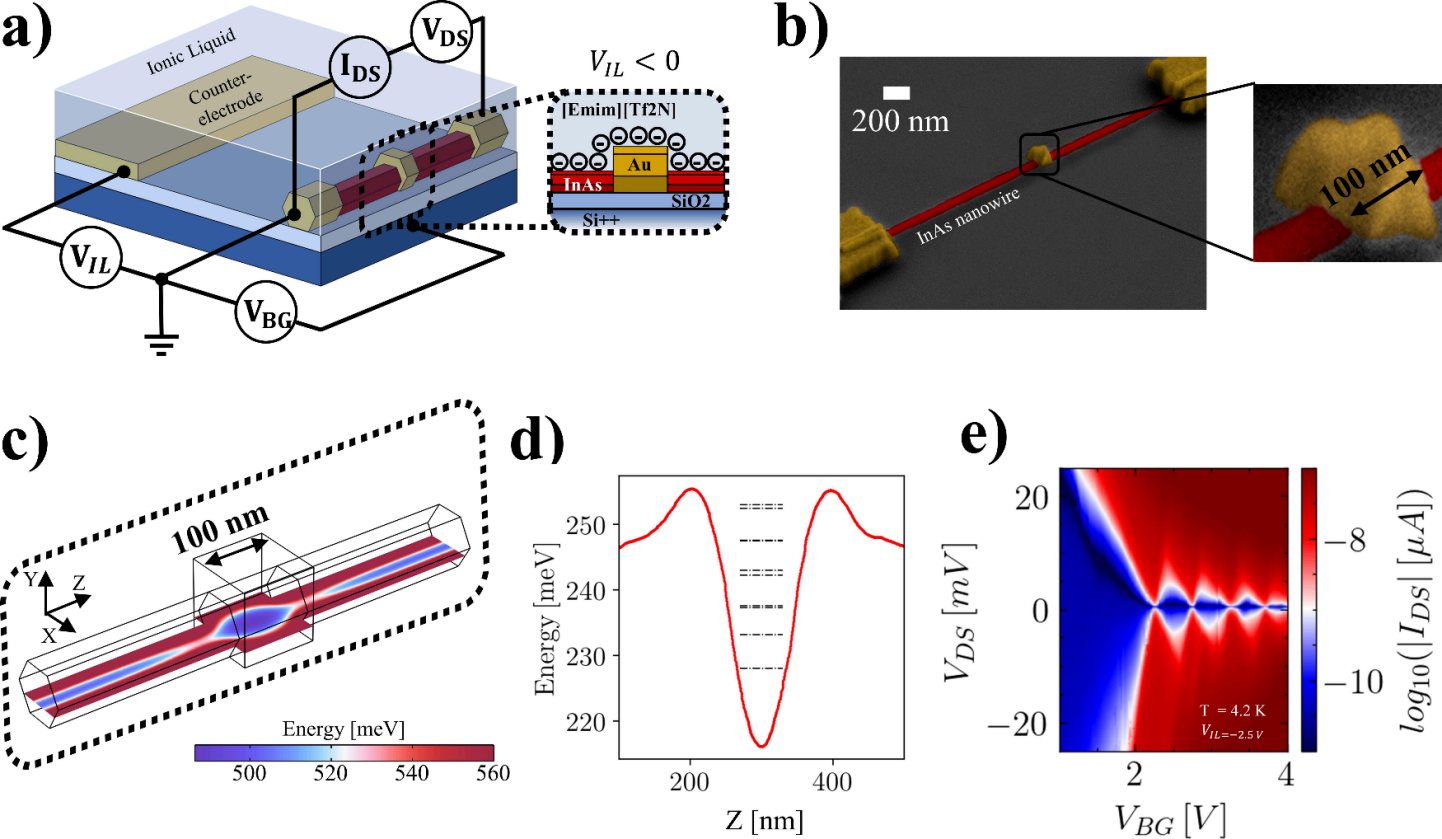
Structure, working principle and electrical transport measurement of a prototypical iontronic quantum dot device developed in this work. (a) Pictorial view of the device implementing a dual-gated structure that combines a conventional back-gate and an ionic-liquid-based gate. The overlay reports the measurement circuital schematic, along with a cross section of the device with a negative voltage *V*_IL_ applied to the counter-electrode. The entire device is immersed in a droplet of ionic liquid, and the ionic distribution is managed via the application of a voltage to a counter-electrode that is coplanar with the nanowire. (b) Tilted scanning electron micrograph of a prototypical device (inset: zoom of the screening patch covering the entire lateral surface of the nanowire). (c) Numerical calculation of spatial energy distribution in a longitudinal cross section of the nanowire. The lowest energy area is formed below the screening patch. (d) Energy profile along the axial direction of the nanowire extracted from the distribution reported in (c). Dashed lines correspond to the calculated discrete energy levels of the confined single particle states enabling the quantum dot transport features. (e) Coulomb blockaded current measurement in the iontronic quantum dot (*T* = 4.2 K, *V*_IL_, freeze = −2.5 V, screening patch width *W* = 100 nm).

It is worth mentioning that these two gates present very different properties and that only the back-gate can be tuned at low temperatures. In fact, at temperatures *T* < *T*_freeze_ (the freezing temperature of [Emim]­[Tf2n] is *T*_freeze_ ∼ 190 K) only a fixed ionic arrangement is available. Consequently, the device is operated in the so-called *set-and-freeze* regime,^26^ meaning that the ionic gate bias *V*_IL_ is set at room temperature and is kept constant during device cooldown. On the contrary, the back-gate can be tuned at low temperatures thus allowing to further tailor the charge-transport properties of the nanostructure by controlling the electrochemical potential of charge carriers in the semiconductor.

The finite-element calculation reported in Figure 1(c) shows the electrostatic potential energy profile in one such experiment (see Supporting Information S.III for further details). It can be noticed that—away from the patch—lower energy regions are defined away from the surface of the nanowire, resulting in elongated channels in which electronic wave functions are nonzero. Below the patch the low-energy region acquires an elliptical shape, and most importantly, a potential well is defined. This feature is more evident in the cut (Figure 1(d)) of this potential landscape as computed along the axis of the nanowire. The potential well-defined below the screening patch can lead to a lower dimensionality system with the appearance of quantized energy levels (indicated by the black dashed lines^27,28^ ); importantly, this potential well is separated from the outlying sections of the nanowire by tunnel barriers. This energetic profile, i.e., a 0D system coupled to charge reservoirs by tunnel barriers, is the typical configuration required for the implementation of a single electron transistor (SET). The formation of a 0D system is confirmed by calculations. The dashed lines represent the lowest single-particle bound-state levels, obtained by solving the Schrödinger equation using the quantum-well potential profile computed from finite-element simulations. Specifically, the calculation uses finite element on-axis 1D potential under the patch, which creates two tunnel barriers connecting the confined region to the reservoirs and yields discrete levels characteristic of a SET. Our experimental data in Figure 1(e) indeed exhibit the typical Coulomb diamond pattern characteristic of SETs in the measured *I*_DS_ at 4.2 K.

Figure 2 reports electrical transport and magneto-transport measurements on the iQD described above at *V*_IL,freeze_ = −1.5 V and *T* = 4.2 K. Figure 2(a) exhibits six Coulomb blockade diamonds, with the first two providing values for the charging energy (*E*_c_ = 12 ± 1 meV) and quantum-level spacing (ϵ = 4.5 ± 0.5 meV). These values are well reproduced by our numerical calculations (see Supporting Information section S.IV) and are comparable to the confinement regimes found in hard-wall InAs/InP QDs.^29^ Interestingly, the first two diamonds are much wider than the subsequent ones. This effect can be ascribed to the interplay between the varying *V*_BG_ and the constant *V*_IL,freeze_. Indeed, in this condition, increasing positive *V*_BG_ values likely lead to a reduced localization of electronic wave functions consistently with the observation of smaller Coulomb blockade diamonds in the *V*_DS_–*V*_BG_ plane.

**Figure 2:**
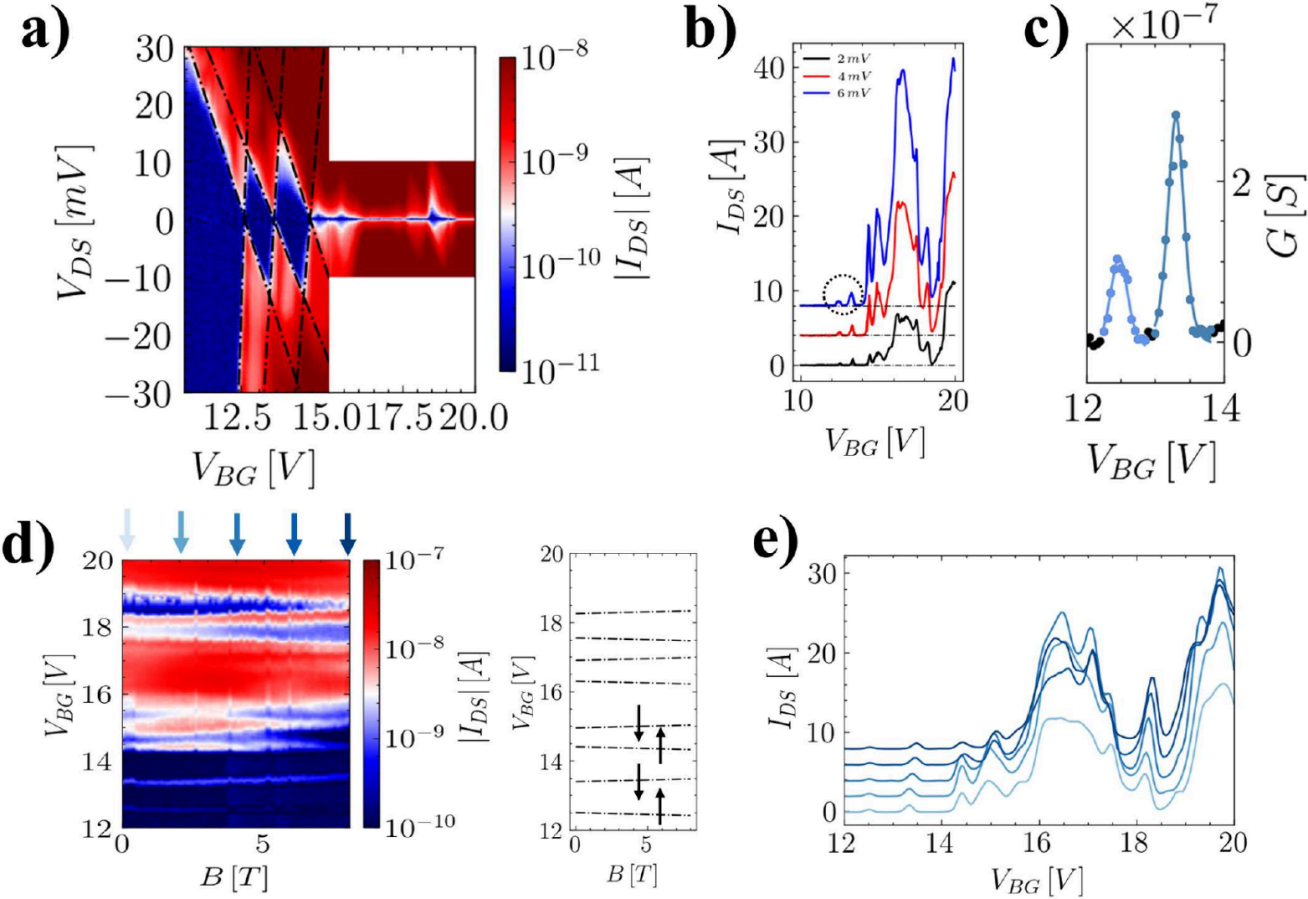
Iontronic quantum dot transport spectroscopy and magneto-spectroscopy (*T* = 4.2 K, *V*_IL,freeze_ = −1.5 V, *W* = 100 nm). (a) Coulomb diamonds at 0 applied magnetic field. Up to 6 Coulomb diamonds are visible, and from the first two diamonds the charging energy and level spacing of 12.0 and 4.5 meV are extracted. (b) Coulomb blockade peaks measured with a bias voltage of 2 mV (black), 4 mV (red) and 6 mV (blue). (c) Best fit on the first two Coulomb peaks measured with an applied bias of 6 mV. From the fit the values for the tunneling rates of the two electrochemical levels are extracted. (d) Electrical transport measurement at fixed *V*_DS_ = 2 mV and in an externally applied magnetic field spanning from 0 to 8 T. The right panel shows the best fit of the linear dependence of the Coulomb peaks with the applied magnetic field, showing that level filling is consistent with Hund’s rule. (e) Line cuts extracted from panel (d) at different values of magnetic field according to the arrows color code.

The impact of the back-gate can also be related to the lower intensity of the first two current peaks with respect to the other Coulomb oscillations. This effect is visible in Figure 2(b) where we show *I*_DS_–*V*_BG_ curves at fixed *V*_DS_ (2, 4, and 6 mV in black, red, and blue, respectively). Two phenomena can contribute to this behavior. On one hand, an increasing carrier density in the injecting portion of the wire is expected for increasing positive *V*_BG_ that leads to an increase in the measured tunnel current, and on the other hand, higher-order iQD levels are involved in the phenomenon with corresponding more transparent tunnel barriers and less iQD energy-level separation. It is worth mentioning here that, even if no detectable signal is measured for low back-gate voltage, we cannot conclude that the first Coulomb diamond observed corresponds to the first iQD level, since the above-mentioned back-gate effect on the whole wire may simply pinch off the wire itself and hinder current flow.

To extract the tunneling rates between the dot and the leads, the conductance of the first two peaks was fitted with the sequential tunneling line shape

$$G=\frac{e^{2}}{h}\frac{\Gamma }{8k_{B}T}\frac{1}{\cosh^{2}\left(\alpha \left(V_{BG}-V_{Th}\right)/2k_{B}T\right)}$$

where Γ is the source/drain tunneling rate (assumed to be equal), α is the back-gate lever arm (extracted from the Coulomb diamond map measurement), *V*_th_ is the device threshold voltage, and *k*_B_ is the Boltzmann constant.^30^ The outcome of the fit is reported in Figure 2(c), and the extracted tunneling rates for the source and drain contacts are Γ_1_ = 2 GHz for the first peak and Γ_1_ = 5 GHz for the second. Interestingly, these values for tunneling rates are coherent with the corresponding values usually reported in the literature for hard-wall QDs, again confirming the efficacy of the iQD scheme.^31^

Magneto-transport measurements are reported in Figures 2(d–f). We report the evolution of Coulomb peaks upon the application of an external magnetic field orthogonal to the NW axis, ranging from 0 to 8 T. For increasing values of the applied magnetic field the peaks drift and their separation increases linearly (Figure 2(f)). This is ascribable to the Zeeman effect on the iQD orbitals, which yields to a *B*-dependence of the peaks’ positions according to $V_{BG_{pk}}=\left(V_{BG_{pk},B=0}+g^{*}\mu_{b}B\right)/\alpha$ for spin up and $V_{BG_{pk}}=\left(V_{BG_{pk},B=0}-g^{*}\mu_{b}B\right)/\alpha$ for spin down,^32^ where g* is the size-dependent effective *g*-factor for InAs and α is the back-gate lever arm extracted from the Coulomb diamonds size. The peak evolution with *B* reported in Figure 2(f) is thus coherent with a standard spin up–down filling sequence of the iQD orbitals. The same value for g* is used to fit all peak maxima, and the value g* = 5.4 ± 0.5 is found—consistent with hard wall InAs quantum dots reported in the literature.^33^

We now analyze the dependence of the sharpness of the observed quantum features on the control parameters employed to define the system, i.e., the width of the screening patch *W* and the gate voltage *V*_IL,freeze_. We used finite element analysis to calculate the electric potential in the nanowire under the electric displacement field *D* generated by ion accumulation on the nanostructure surface. We consider the eigenstates of the confined electrons and the effect of the screening-patch width on the quantum features of the iQD. Figure 3 reports the main results of our investigation. Specifically, Figures 3(a,b) show the normalized ground-state electronic wave functions for several values of the confinement field at a fixed value of the patch width (*W* = 100 nm). Figures 3(c,d) report the corresponding results calculated with a fixed value for the confinement field (*D* = 0.0046 C/m^2^) for different values of the screening-patch width. These results show that the confinement field has a much stronger effect on the spatial extension of the electronic wave function when compared to the effect of the patch width, suggesting that the applied liquid-gate voltage is the main driving force toward the definition of the confined 0D system. To probe the combined effect of *W* and *D* on the quantum features of the system, the energy difference between the first two states was computed in the parameter space spanned by these quantities (Figure 3(e)). Calculations reported in Figures 3(a–d) were obtained using a simplified approach, designed to assess whether the confinement field or the patch width plays the dominant role in setting the spatial extent of the wave function. As discussed later in this work, the model has a limited validity and is suitable for comparing these two parameters, while it is insufficient to account for all experimental findings. In particular, the model does not consider some important mechanisms, such as the depletion of the leads induced by the confinement field generated by the ionic-liquid gate and crosstalk between the left/right potential barriers. These mechanisms are believed to play a relevant role in explaining the quantum dot stability diagram reported in Figure 4.

**Figure 3:**
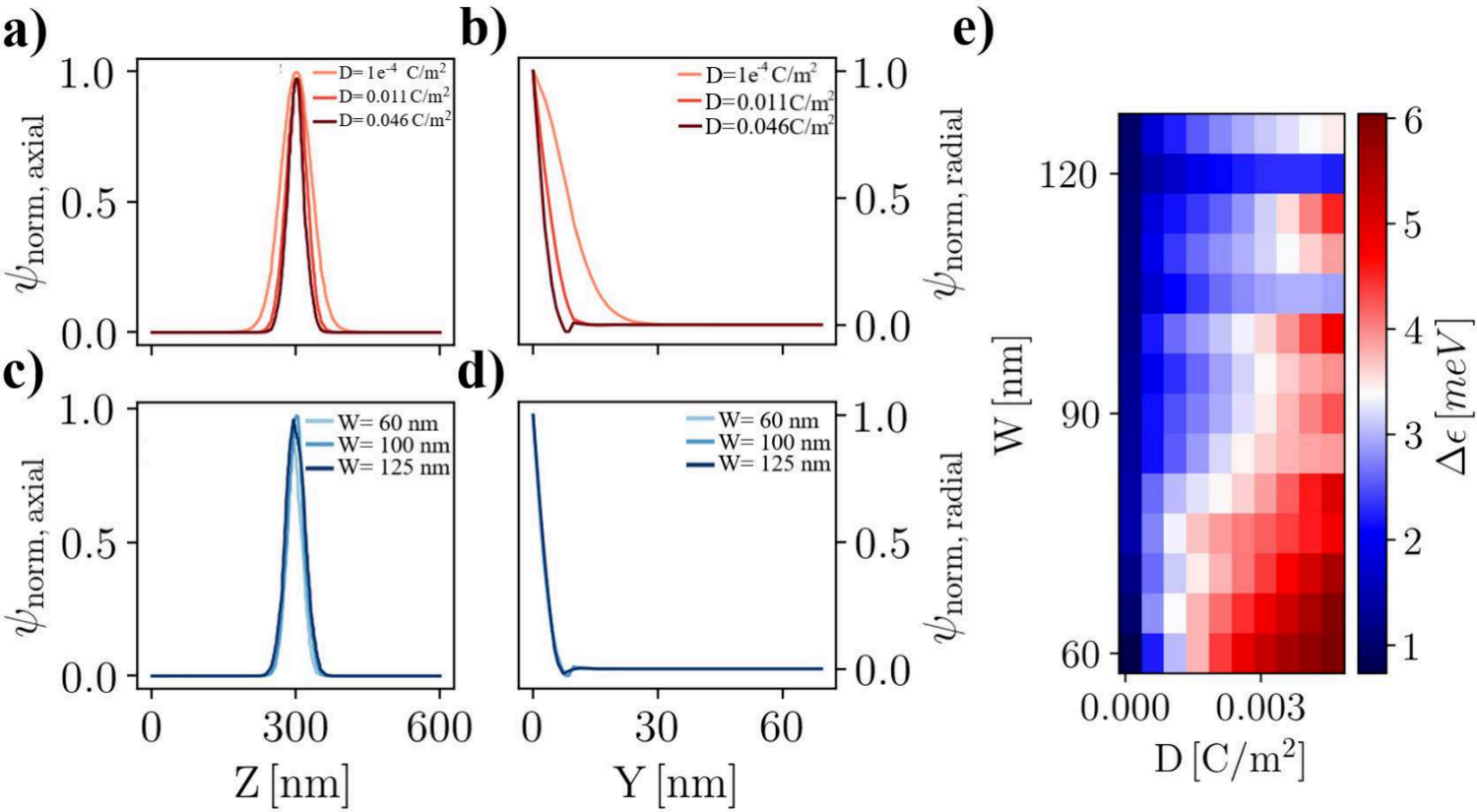
Spatial profiles of the iontronic quantum dot ground state wave function, calculated by varying the screening patch width *W* and confinement field value *D*. (a, b) Normalized ground state wave function line shape along the axial (a) and radial (b) direction of the nanowire calculated for different values for the confinement field, showing that this parameter has a strong impact on the spatial extension of the quantum state. (c, d) Corresponding calculations of the ground state wave functions for several values of screening patch widths, showing that the effect on the wave function’s spatial extension is strongly suppressed. (e) Quantum level spacing between the first two confined levels in the iontronic quantum dot Δϵ as a function of *W* and *D*.

**Figure 4:**
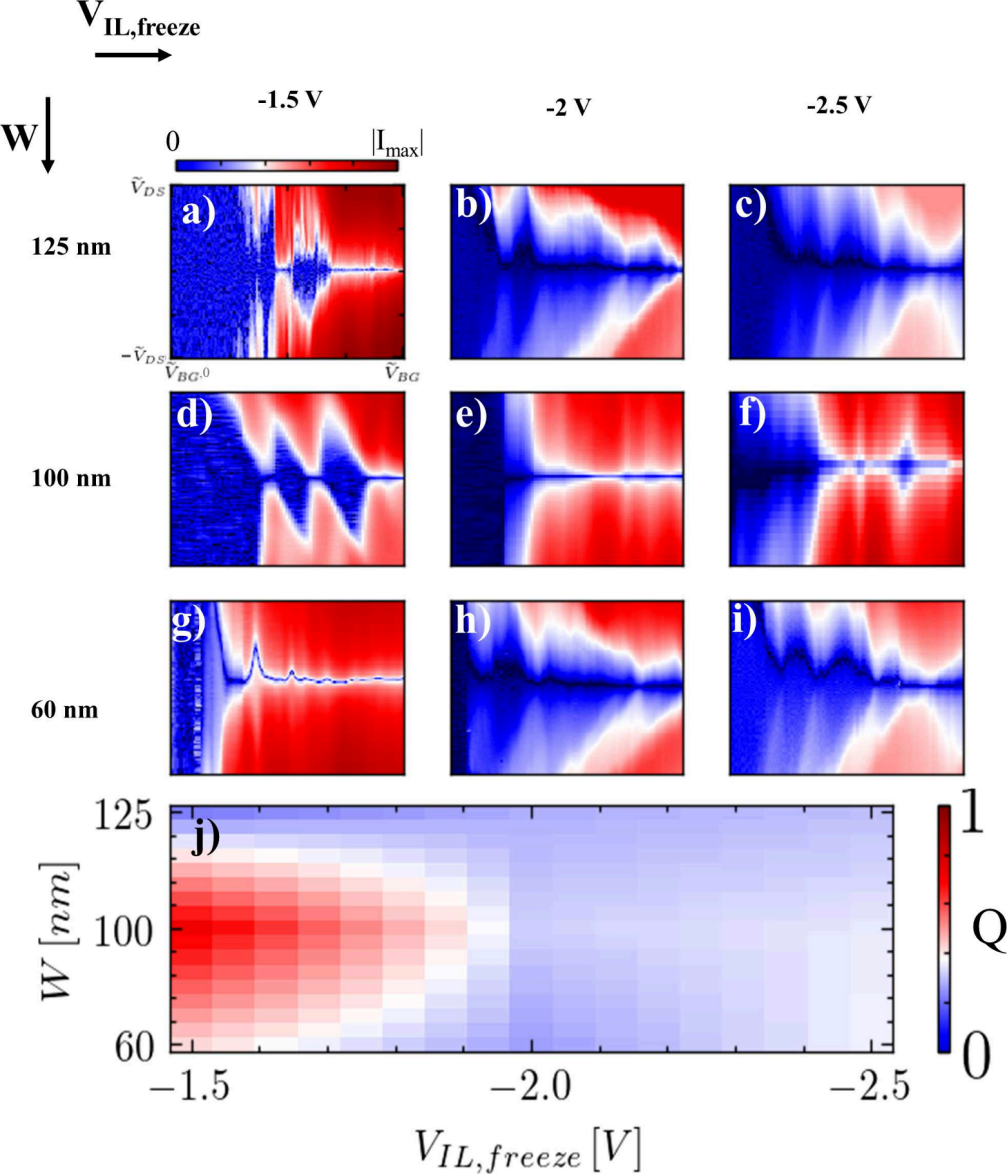
Experimental evolution of the iontronic quantum dot stability diagram and figure of merit *Q* with the screening patch width *W* and confinement voltage *V*_IL,freeze_. (a–i) Coulomb blockade diamonds for 3 different values of the screening patch width (125 nm, 100 nm, 60 nm) and confinement voltage (−1.5 V, −2 V, −2.5 V). Each measurement reports the absolute value of current flowing in the quantum dot up to the maximum measured value within a range of bias and back-gate voltages allowing to observe at least two Coulomb diamonds. (j) Iontronic quantum dot quality factor in the parameter space spanned by screening patch width and confinement voltage.

To compare these numerical results with experimental data, several iQDs were realized with different patch widths *W* and confinement fields *D*, as reported in Figure 4. Devices with screening patch widths of 60, 100, and 125 nm were fabricated on the same nanostructure (see Supporting Information S. V for details regarding device fabrication) and tested by fixing the freezing liquid-gate voltage to −1.5 V, −2 V and −2.5 V. These ionic-liquid gate voltage biases were selected to lie within the electrochemical window of the ionic liquid [Emim]­[TF2n], thereby minimizing the risk of electrochemical side reactions in the electrolyte and at the device interfaces. The results are shown in Figures 4(a–i), reporting Coulomb blockade maps for each of the investigated configurations in a *V*_DS_ range $\left(-{Ṽ}_{DS},{Ṽ}_{DS}\right)$ and *V*_BG_ range $\left({Ṽ}_{BG,0},{Ṽ}_{BG}\right)$ focusing on multiple Coulomb diamonds. Noticeably, although Coulomb blockade features are visible in all data sets, their quality and sharpness are found to strongly vary with respect to *W* and *V*_IL,freeze_. Specifically, a trend is noticeable going from the weaker (*V*_IL,freeze_ = −1.5 V) to the stronger confinement regime (*V*_IL,freeze_ = −2.5 V), showing that, in the latter case, the quality of the quantum structure is decreased. On the other hand, a similar trend for the screening patch width *W* is not evident. Indeed, the best iQD was observed in the case of *W* = 100 nm and *V*_IL,freeze_ = −1.5 V. To quantify this observation, each Coulomb blockade map was analyzed by extracting a figure of merit parameter *Q*, computed as the product of the first-diamond height with the total number of Coulomb diamonds observable before reaching the continuum, normalized to 1. The experimental results were interpolated to produce the map reported in Figure 4(j). Noticeably, the results of the experiments are consistent with the numerical data, demonstrating that the intensity of the confinement field has a more pronounced impact on the quality of the QD compared to the width of the screening patch. However, a difference is observed concerning the trend of the quality factor with respect to *W* and *D*: the experimental findings reported in Figure 4(j) allow identification of a hot spot at the lowest confinement strength and intermediate patch width. In contrast to numerical simulation, which suggested a weak influence of the patch width on the wave function, the experimental results demonstrate an important role of this parameter in determining the iQD’s stability diagram, along with the confinement field. As mentioned in the discussion of Figure 3(a–d), the model does not take into consideration the depletion of the leads acting as reservoirs for the quantum dot and the energy profile of the confinement barriers. Indeed, for more negative liquid gate potential, the leads are much more depleted compared to the configurations in which the system is frozen with a more positive applied liquid-gate voltage. In this condition, a stronger back-gate voltage is needed to compensate for the depletion induced by the ionic liquid gate and the electrical conduction through the iQD involves higher-order, energetic and less-separated states. Additionally, when smaller screening patch widths are concerned, a certain amount of interbarrier crosstalk can be envisioned, leading to detrimental effects on the quantum confinement of charge carriers. Ultimately, the sweet spot for iQD SET operation corresponds to a configuration characterized by relatively weak confinement (i.e., *V*_IL,freeze_ = −1.5 V) and by the intermediate width of the screening patch. Specifically, the latter should be long enough to avoid any crosstalk between the tunnel barriers and short enough to enable the potential well defining the quantum dot to be deep and exhibit well-spaced levels, as in the case for *W* = 100 nm.

Finally, we investigate the flexibility of our approach toward the realization of multiple QD systems. To this aim, a device fabricated with a single InAs nanowire featuring a series to two iontronic quantum dots was fabricated, as shown in Figure 5(a). Here, two 100 nm wide screening patches were fabricated on the nanowire so that the distance between them was large enough (1 μm) to prevent any coherent transport or tunneling between the two quantum systems. The rationale of this device is to measure the electronic transport through a system, as represented in Figure 5(b). Since the two quantum dots are decoupled, the measurement of two identical quantum dots in series would be expected. Indeed, electrical transport is enabled when the electrochemical potentials of the two quantum dots, e.g., μ_n,LQD_ and μ_n,RQD_ in Figure 5(b), are aligned. Since the two iQDs are nominally identical (the two screening patches of the same width are fabricated in a single fabrication step together with the ohmic contacts and the same droplet of ionic liquid is covering both structures) the pattern observed in quantum transport for the system under analysis is expected to result from the overlap of two identical single iQD patterns. Panels in Figure 5(c–e) report bias spectroscopy measurement of the device (4.2 K, *V*_IL,freeze_ = −1.5 V). Specifically, Figure 5(c) shows a series of nearly identical pairs of Coulomb diamonds in the Coulomb blockade map. The nonideal overlap of the electrochemical level spectra of the two dots is ascribable to differences in the local electrostatic landscape in the two nanowire sections where the QDS are formed, which may be caused by inhomogeneities in the surface states by impurities in the semiconductor crystal lattice, causing a small rigid shift between the energy spectra of the two QDs. Figure 5(d) allows us to observe that the paired Coulomb peaks overlap and a fit of the tunneling rates for the first two peaks (as highlighted in Figure 5(e)) returns Γ_1_ = 5.4 ± 0.5 GHz and Γ_2_ = 5.7 ± 0.5 GHz for the first and second peaks, respectively. These values are consistent with each other, suggesting that they are coming from corresponding levels in two identical QDs. The picture presented here is further confirmed by the magneto-transport measurements shown in Figure 5(f), where the paired peaks evolve in a parallel fashion in an externally applied magnetic field (from 0 to 8 T), reflecting that they refer to parallel spins isolated in the two iQDs.

**Figure 5:**
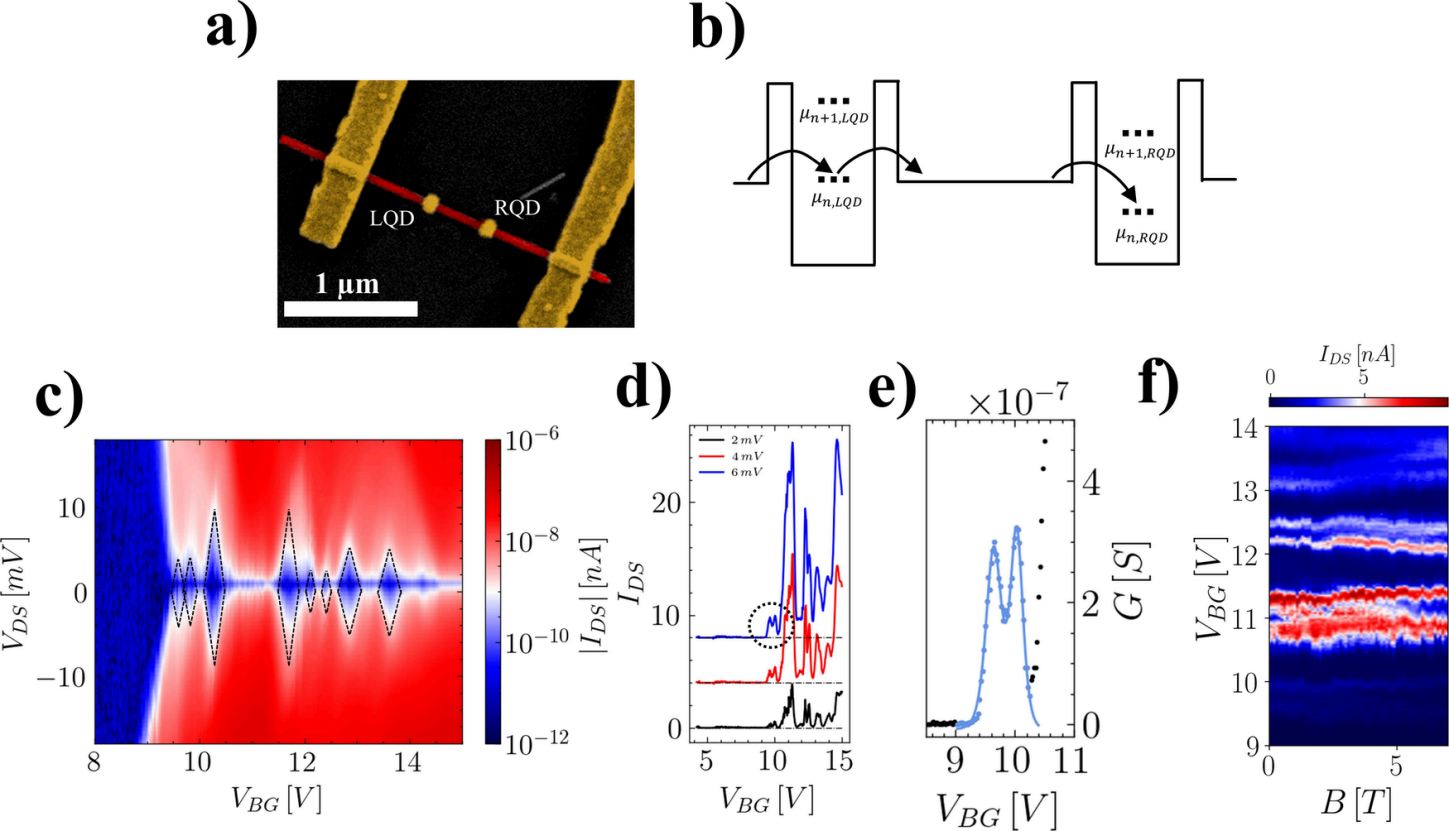
Electrical transport and magneto-transport measurements of two identical iontronic quantum dots fabricated in series. (a) Scanning electron micrograph of a prototypical device: two 100 nm wide screening patches 1 μm away from each other are fabricated on the same nanowire. (b) Pictorial energy diagram of the series quantum dots at 0 applied bias voltage, evidencing identical energy spectra. (c) Bias spectroscopy measurement revealing pairs of Coulomb diamonds with comparable size. (d) Coulomb peaks measured for *V*_DS_ = 2 mV (black), 4 mV (red) and 6 mV (blue). Paired peaks with the same height are observed. (e) Best fit of the first two Coulomb peaks measured for *V*_DS_ = 6 mV. (f) Coulomb peaks measured with an externally applied magnetic field from 0 to 8 T, showing that paired peaks evolve in a parallel fashion.

In conclusion, a novel paradigm of engineering quantum nanostructures has been demonstrated, leading to the realization of the iQD. This device concept exploits the unprecedented strong electric fields accessible with ion-gating to reduce the dimensionality of an electron system and define high-quality 0D systems by resorting to a single-step fabrication process and homogeneous nanowires. Negative ions are accumulated on the electrolyte/nanowire interface, except for a small segment of the semiconductor where a thin patch is fabricated and leads to the quantum confinement of electrons. The system is cooled in this configuration, and at low temperatures the ionic arrangement is frozen in place, yielding high stability to the confinement potentials. At low temperatures, a back-gate is employed to control the resulting iQD. Bias spectroscopy and magneto-transport measurements have been performed on this new system, finding that the quality and stability of the confinement enabled by ion gating can go on par with hard-wall QDs. To demonstrate the reproducibility of the approach, a device implementing two nominally identical iQDs in series was realized and characterized, showing that a virtually arbitrary number of high-quality QDs can be engineered on a single-crystal semiconductor. This work opens the way to an entirely new class of quantum devices exploiting the favorable properties of ion gating to implement tailored quantum wave functions for charge carriers in semiconductors. The developed platform offers a high level of engineerability, allowing for the optimization of ideally every component; it is material-agnostic and applicable to every semiconductor system. We underline the relevance of this result in the context of quantum technologies since it opens the way to the realization of on-demand high-performance quantum systems featuring ease of fabrication and favorable electrical transport properties, with a broad range of potential applications. Our approach combines a single lithography step with an ionic-liquid electric-double-layer (EDL) “set-and-freeze” operation to realize hard-wall-like confinement and stable Coulomb blockade spectroscopy without multilayer metal/oxide gate stacks or dense multigate tuning. This simplification accelerates the prototyping of single and multiple quantum dots for quantum transport applications. In fact, in CMOS gate-defined architectures, state-of-the-art devices leverage overlapping gates and high-quality dielectrics optimized in foundry processes, effective for scale-out but fabrication-intensive for lab settings.^34^ By contrast, our one-step flow preserves the spectroscopic quality while drastically reducing process complexity. Also compared to epitaxial hard-wall nanowires (e.g., InAs/InP axial heterostructures)^35^ where confinement is fixed at growth, our dots do not require heterostructure epitaxy and remain postfabrication tunable through EDL modulation in set-and-freeze operation, retaining the key advantages of hard-wall-like spectra with added reconfigurability.

## Supplementary Material



## Notes

S. Methods, details on nanowire growth and device fabrication; S. I., electrical transport of InAs nanowires without confinement fingers; S. II., confinement fields calculation; S. III., electrostatic confinement potential in ion gated nanowires; S. IV., quantum level spacing calculation: S. V., impact of different finger sizes and ionic gate applied voltage; S. VI., Coulomb diamonds measurements with BB ≠ 0 (PDF)
