# Supplementary material for: The Iontronic Quantum Dot
Source: Nano Lett. 2025 Nov 27;26(2):691–8. doi: 10.1021/acs.nanolett.5c03173 (PMC12833871; doi:10.1021/acs.nanolett.5c03173)
Supplement: Supplementary file 1 [file nl5c03173_si_001.pdf]

The Iontronic Quantum Dot. Supporting Information

Domenic Prete[1], Valeria Demontis[1], Valentina Zannier[1], Lucia Sorba[1], Fabio Beltram[1], and Francesco Rossella[2]

[1]NEST, Scuola Normale Superiore and Istituto Nanoscienze-CNR, Piazza San Silvestro 12, I-56127, Pisa, Italy
[2]Dipartimento di Scienze Fisiche, Informatiche e Matematiche, Università di Modena e Reggio Emilia, via Campi 213/a, 41125 Modena, Italy

Section S. Methods

NANOWIRE GROWTH. Nominally undoped InAs nanowires were grown by chemical beam epitaxy (CBE) in a Riber Compact-21 system on InAs (111)B substrates by Au-assisted growth [Gomes2015]. A thin (0.5 nm) Au film was deposited on the substrates at room temperature by thermal evaporation in a metallization chamber equipped with quartz crystal thickness monitor. The substrate was then introduced in the CBE chamber and a thermal annealing (20 min at 440 +/- 10 °C under As flux) was carried out to trigger Au dewetting and nanoparticle formation. After lowering the temperature to 390 +/- 10 ° C, the nanowire growth was started. We used trimethylindium (TMIn) and tert-butylarsine (TBAs) as metalorganic precursors, with line pressures of 0.6 and 1.5 Torr, respectively. After 2 h the growth was stopped, and the sample cooled down to 150 ° C under As flux. Morphological characterization of the grown nanowires was performed using a Zeiss field-emission SEM operated at 5 kV.

DEVICE FABRICATION. As-grown InAs nanowires were mechanically detached from the InAs substrate via sonication in isopropyl alcohol (IPA) and subsequently a droplet of the suspension was cast on top of a Si++/SiO_2 fabrication substrate with bonding pads pre-patterned via UV lithographic techniques. Scanning electron micrographs of just-deposited nanowires were taken in order to align the computer assisted design of devices to specific nanowire (random) orientations on the substrate. Prior to electron-beam lithography (EBL), substrates were spin-coated with PMMA-based resist (Allresist AR-P 679-04) at 4000 rpm for 1 min and resist was baked at 170 ° C for 1 min and 30 s. After EBL, the resist was developed and a passivation step in a standard NH_42S_X solution performed. This step is required in order to etch the native oxide forming on the surface of InAs nanowires and allows to achieve good ohmic contacts for transport measurements. After passivation, a Ti/Au bilayer (10/100 nm) was thermally evaporated on the substrate, which was then immersed in hot acetone (50 ° C) for lift-off. After lift-off, devices were wire bonded to commercial dual-inline packages used to plug the final device in the measurement setup.

Section S. I. Electrical transport of InAs nanowires without confinement fingers.

In order to rule out the occurrence of any random quantum dots not generated by the presence of the confinement finger as shown in the main text, control devices based on nominally identical InAs nanowires are fabricated without the presence of any finger protecting a thin section of the nanostructure. Such devices are fabricated simultaneously to the iontronic quantum dots on the same

fabrication substrate, and the nanowires on which quantum dots are defined and those to perform the control measurements are randomly chosen during the CAD design process.

The control devices are embedded in the same polarized ionic liquid droplet which is employed to acquire the data shown in the main text, and electrical transport measurements are performed at 4.2 K with the same experimental setup. As shown in Figure S1, the absence of the confinement finger results in the complete absence of any Coulomb blockade features in the electrical response of the nanowire, suggesting that the only factor which is providing quantum features and the definition of the 0D system thoroughly described in the main text is, indeed, the presence of the confinement finger.

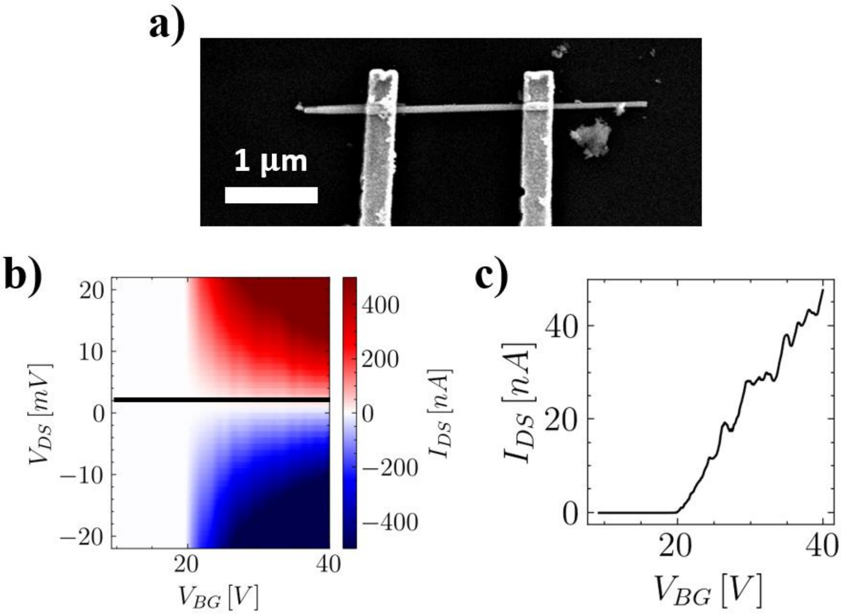

Figure S1: a) Scanning Electron Micrograph of a fabricated device based on an InAs nanowire nominally identical to the ones employed to realize iontronic quantum dots. The device architecture is identical to the iontronic quantum dot one, except for the absence of the confinement finger. b) Measurement of the current flowing in the nanowire at 4.2 K, sweeping the back-gate voltage and the bias voltage. c) Line cut of the previous map for a fixed bias voltage V_{DS} = 2 mV.

Section S. II. Confinement fields calculation

To evaluate the electrostatic potential distribution in the system under analysis, finite element modelling was employed to calculate the displacement fields generated at the ionic liquid/nanowire interface in a geometry similar to the experimental one. The calculation is performed using the COMSOL Multiphysics software [1], specifically the Transport in Concentrated Species and Electrostatics modules, coupled via Space Charge Density Coupling and Potential Coupling. The calculation, shown in Figure S2, is performed on a 2D model of the bulk of the ionic liquid with two Dirichlet boundary conditions: a voltage applied to the top boundary of the domain (i.e., the applied ionic gate voltage) and 0 voltage set on the interface of a hexagonal terminal (corresponding to the surface of the nanowire when the ionic liquid is polarized during cooldown procedures).

The following system of equations is solved:

$$\begin{cases} -\nabla \cdot \left(\rho D_i^f \nabla \omega_i + \dfrac{\rho \omega_i D_i^f \nabla M_n}{M_n} + \dfrac{\rho \omega_i z_i D_i^f}{RT} F \nabla \phi \right) = 0 \\ \nabla^2 \phi = \dfrac{F}{\epsilon_0 \epsilon_r} \sum_j z_j c_j \end{cases}$$

Where ρ is the material's density, D_i the diffusion coefficient of the i-th species (i = 0 begin the anion, i = 1 the cation), ω_i the mass fraction of the i-th species, z_i its charge, c_i its concentration and M_n is defined as:

$$M_n = \left(\sum_j \frac{\omega_j}{M_j} \right)^{-1}$$

M_j being the molar mass of the j-th species.

The result of such calculation is reported in Figure S2. Here, the main properties of the formation of electric double layers in the electrolyte are obtained [3], namely an abrupt potential drop in the immediate proximity of the electrodes related to the abrupt change of ionic species local concentrations, while the bulk of the electrolyte remains in a neutral state where the potential drop is zero and the concentration of the ionic species are equal.

This calculation allows us to estimate the values for the displacement fields for several values of applied gate voltages: these values will be employed in a subsequent calculation as a boundary condition to compute the electrostatic potential in the semiconducting nanowire which will define the potential term in the Hamiltonian for the charge carriers in the nanostructure. Furthermore, values for the electric fields applied at the ionic liquid/nanowire interface of the order of MV/cm are found, consistent with values reported in literature for similar applications [2].

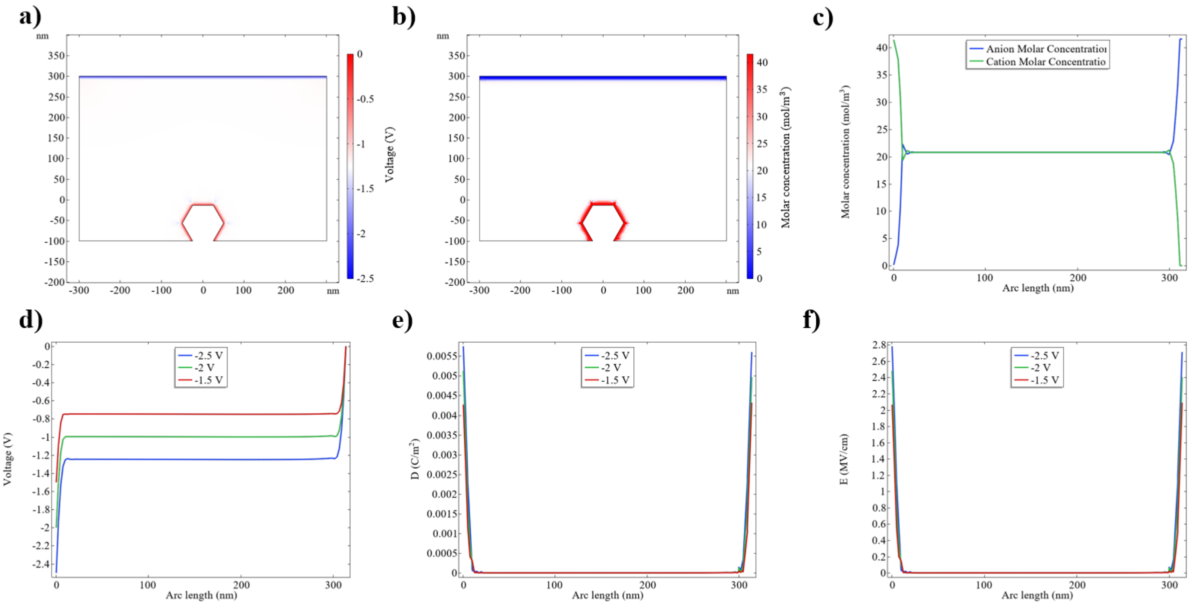

Figure S2: Calculation of the applied displacement and electric fields at the nanowire interface with ion gating. a) Voltage vs position in a geometry resembling the experimental one. -2.5 V are applied at the top boundary of the domain, while the nanowire (hexagonal cross-section) is grounded. The potential drop mainly happens in the proximity of the interfaces and the bulk of the electrolyte is equipotential. b) Molar concentration of anions in the electrolyte calculated in the configuration described previously. c) Cross section in the y direction corresponding to x = 0. As expected, the molar concentrations of cations and anions follow opposite behaviours, and the anions are accumulated at the nanowire/electrolyte interface. d) calculated potential drop for several values of gate voltages (-2.5 V, -2 V and -1.5 V). The potential drop is shown to occur in the proximity of the electrode. e) Electric displacement field on the cut along the x = 0 direction for different values of applied gate voltage. The maximum value is 0.0057 C/m^2 for -2.5 V at the nanowire's surface. f)

Resulting electric fields applied by means of ion gating. The order of magnitude (MV/cm) is consistent to the values reported in literature [2].

Section S. III. Electrostatic confinement potential in ion gated nanowires

Once the displacement field applied by the ions at the surface of the nanowire is known, it can be used as a boundary condition to compute the electrostatic potential in the entire domain constituted by the nanowire and a finger covering five of its facets. This system is reminiscent to the devices fabricated in this work, since the confinement finger covers the entire nanowire, except for the facet which is laying on the fabrication substrate.

The equation solved in this case is:

$$\nabla \cdot \boldsymbol{D} = \rho_v$$

With the boundary conditions of a displacement field applied on all the facets except with the bottom one, which instead is grounded, reminiscent of a configuration in which the ionic liquid is polarized to set the confinement potential in the nanowire and the back-gate is set to 0.

$$\begin{cases} \boldsymbol{n} \cdot \boldsymbol{D} = \boldsymbol{n} \cdot \boldsymbol{D_0} & \text{top facets} \\ \boldsymbol{n} \cdot \boldsymbol{D} = 0 & \text{bottom facet} \end{cases}$$

Here, $\boldsymbol{n}$ is the normal vector to the nanowire facet and $\boldsymbol{D_0}$ is the displacement field generated by the ions of the polarized ionic liquid.

Solving this equation allows to evaluate the electrostatic potential ϕ in the entire domain, as shown in Figure S3. Here, several plane cuts in the domain are reported varying the height of the cut. Interestingly, there is a smooth variation in the potential profile in the nanowire by varying the z coordinate, i.e., the difference between confinement in the leads (that is, the nanowire sections outside of the quantum dot region) and below the confinement finger gets progressively sharper. For this reason, we believe that in our architecture the iontronic quantum dot transport features observed in our work are coming from electrical transport in the region of the semiconductor near the bottom facet of the nanowire, where the potential profile confining the electrons is smoother and giving rise to lower potential regions.

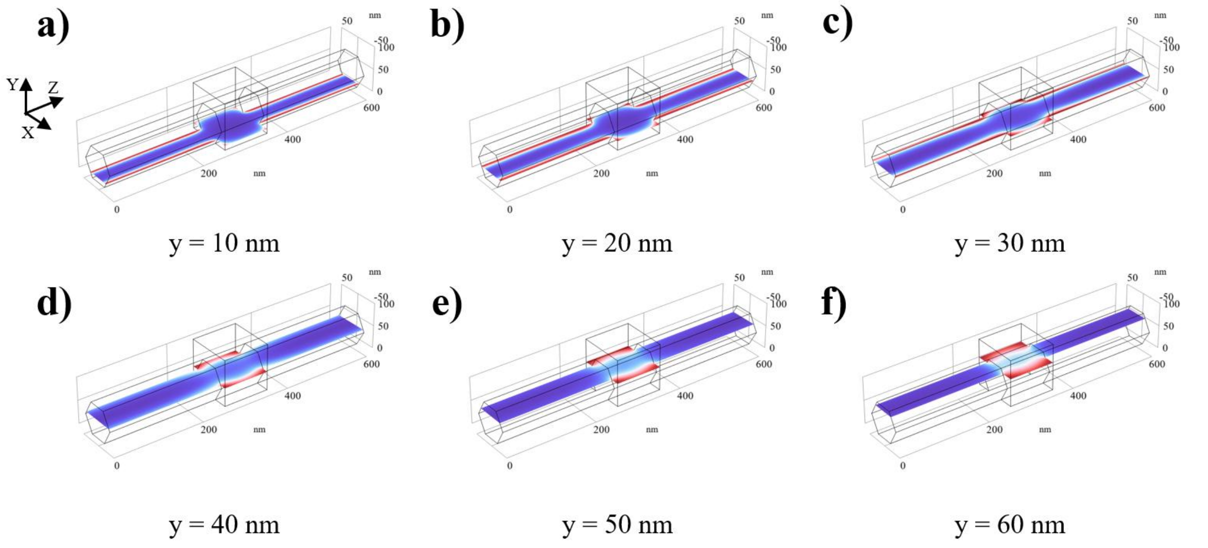

Figure S3: Computed electrostatic confinement potential in the nanowire along the axial direction, computed for several values of the z coordinate. a) 10 nm. b) 20 nm. c) 30 nm. d) 40 nm. e) 50 nm. f)

60 nm. Going towards higher z coordinates, a diminished confinement effect in the leads coupled with a stronger confinement effect in the quantum dot area below the finger are evident.

Section S. IV. Quantum level spacing calculation

For the calculation of the quantum features of the system, the electrostatic potential computed as described in the previous section is used as the potential term in the Hamiltonian for the charge carriers to solve the Schrödinger equation:

$$\sum_{n=1}^{N} H_{mn}\psi_n^j(\boldsymbol{r}) = E_j\psi_m^j(\boldsymbol{r})$$

Where in the kinetic term of H_{mn} the effective mass of InAs $m^* = 0.023m_e$ is considered, and the potential term for the electrons is calculated as $V = -e\phi$, where ϕ is the electrostatic potential calculated as described in section S. III and e is the electron charge.

This allows to compute the energy levels and wavefunctions for electrons in the entire domain, as reported in Table 1, showing the first 10 energy levels of the system computed for a finger width of 100 nm and a displacement field of 0.0046 C/m^2, coming from an applied liquid gate voltage of -1.5 V. As discussed in the main text, the quantum spacing $\Delta\epsilon = E_1 - E_0 \sim 5\ meV$ is found to be consistent with the experimental value (4.5 $\pm$ 5 meV), and the subsequent levels are found to be 2-fold degenerate, ascribable to the specific symmetry of the system as described in the main text.

**Level #**	**E [meV]**
0	228.00
1	233.21
2	237.26
3	237.63
4	242.27
5	243.01
6	247.44
7	247.58
8	252.42
9	252.97

Section S. V. Impact of different finger sizes and ionic gate applied voltage.

The impact of confinement finger width and freezing ionic liquid gate voltage on the iontronic quantum dot quality was investigated and assessed. To do so, several devices were fabricated implementing different widths for the confinement gates (60, 100 and 125 nm) and cooled down simultaneously with a global ionic liquid gate voltage, in order to avoid any difference induced by the cooldown procedures, if any. Furthermore, different confinement finger widths were implemented on the same nanowire while keeping fixed the total channel length between ohmic contacts, as

shown in Figure S4(a). This was realized in order to minimize any difference in device operation coming from different nanowires.

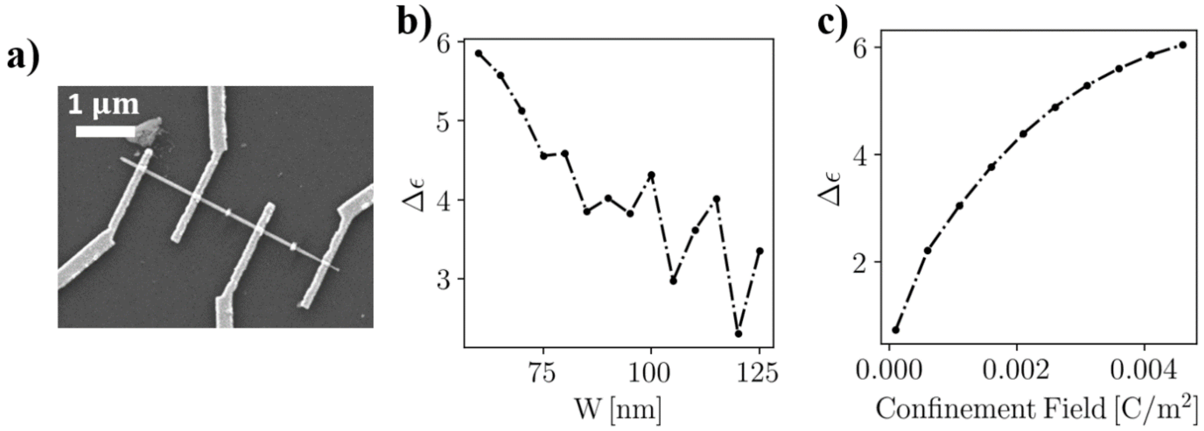

Figure S4: Impact of finger width and ionic gate voltage on device properties. a) Example of fabricated devices with confinement finger featuring different widths. In order to reduce any spurious effects coming from slight differences from nanowire to nanowire, the same nanostructure is employed to probe the quality of the iontronic quantum dot for different values of confinement finger width. b) Impact of confinement width on the calculated quantum level spacing between the first two levels. c) Impact of the applied ionic gate value on the calculated quantum level spacing between the first two levels.

Furthermore, the impact of finger widths and freezing gate voltage were assessed by means of the finite element analysis framework developed for this work and described in sections S. II, III and IV. The results are reported in Figure S4(b)-(c), showing the difference between the first two energy levels ($\Delta\varepsilon$) depending on the finger width and liquid gate voltage, respectively. While the impact of the confinement field on $\Delta\epsilon$ is rather smooth, the confinement finger width has a much more irregular impact for W > 75 nm.

Section S. VI. Coulomb diamonds measurements with $B \neq 0$

Figure S5 reports the Coulomb blockade maps measured on a single iontronic quantum dot device and two in-series quantum dots at 4.2 K with a constant magnetic field B = 8 T applied orthogonally with respect to the nanowire axial direction. While the main features of the iontronic quantum dot are preserved also when a constant magnetic field is applied, in both cases the relative size of the first two diamonds is changed compared to the B = 0 T case: indeed, the first diamond is now taller. This effect may be ascribable to the first level being shifted downwards in energy due to its spin up filling, causing it to be in the leads' pinch-off region and this not accessible with this experimental configuration.

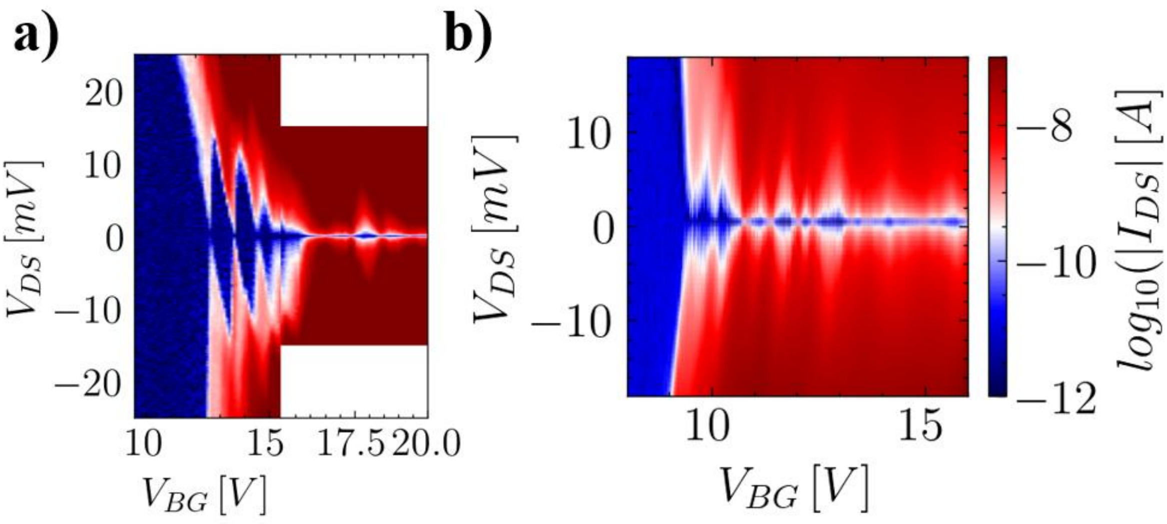

Figure S5: Coulomb blockade measurements at B = 8 T for a) a single iontronic quantum dot device and b) a two in-series iontronic quantum dots devices.

References

[1] COMSOL Multiphysics® v. 6.1. www.comsol.com. COMSOL AB, Stockholm, Sweden.

[2] Bisri, S. Z., Shimizu, S., Nakano, M., Iwasa, Y., Adv. Mater. 2017, 29, 1607054. https://doi.org/10.1002/adma.201607054

[3] Maxim V. Fedorov and Alexei A. Kornyshev Chem. Rev. 2014, 114, 5, 2978–3036, https://doi.org/10.1021/cr400374x
